# The role of food during oncology treatment: perspectives of cancer patients, caregivers and healthcare professionals

**DOI:** 10.1007/s00520-024-08469-4

**Published:** 2024-04-22

**Authors:** Dominika Adamczyk, Dominika Maison, Stella Lignou, Omobolanle O. Oloyede, Miriam Clegg, Lisa Methven, Carol Fairfield, Margot Gosney, Maria José Hernando, Javier Amézaga, Mercedes Caro, Itziar Tueros

**Affiliations:** 1 Faculty of Psychology, University of Warsaw, Stawki 5/7, 00-183, Warsaw, Poland; 2 Department of Food and Nutritional Sciences, Harry Nursten Building, University of Reading, Pepper Lane, Whiteknights, Reading, RG6 6DZ UK; 3 Department of Nutrition, Food and Exercise Sciences, Dorothy Hodgkin Building, University of Surrey, Stag Hill, Guilford, GU2 7XH UK; 4 School of Food and Nutritional Sciences, University College Cork, Cork, Ireland; 5 School of Psychology and Clinical Language Sciences, University of Reading, Reading, UK; 6 AZTI, Food Research, Basque Research and Technology Alliance (BRTA), Parque Tecnológico de Bizkaia, Astondo Bidea, Edificio 609, 48160 Derio-Bizkaia, Spain

**Keywords:** Social role of eating, Cancer patients, Qualitative study, Nutrition, Nutrition support, Malnutrition, Food intake, Taste and smell alterations

## Abstract

**Purpose:**

Many cancer patients have problems eating which are usually connected to taste and smell alterations due to side effects of cancer treatment. These problems have consequences both in terms of malnutrition and reduced quality of life. In order to explore social and psychological consequences of eating problems in cancer patients, qualitative interviews were conducted with cancer patients, their caregivers and healthcare professionals.

**Methods:**

The study was conducted in three European countries (Poland, Spain and the UK) that differed in culture, oncology care approaches and availability of nutritional products targeted to cancer patients in the market.

**Results:**

Differences in the social role of eating between the three European countries were observed which subsequently influenced the impact of eating problems for cancer patients in these countries. Furthermore, the study found that problems with food affect not only the quality of life of cancer patients, but can also distress their caregivers, who are often unable to cope with such food-related problems. In addition, the study showed that commercially available nutritional products for cancer patients focus on nutritional value but tend to neglect an important aspect of eating, which is the enjoyment of food, both individually and socially.

## Introduction

Cancer is one of the leading causes of death worldwide. According to the Global Cancer Observatory, in 2020 there were 19.3 million new cases and 9.9 million cancer-related deaths worldwide [1]. One of the main challenges for cancer patients undergoing treatment is to prevent, mitigate or treat malnutrition. Cancer-related malnutrition is highly prevalent in patients undergoing cancer treatment and varies from 20 to 70% depending on cancer site and stage [2–4]. On the other hand, although not all patients suffer from malnutrition, many do experience a reduction in their daily food intake, as they often suffer from adverse side-effects such as nausea-vomiting, loss of appetite, sores in mouth, difficulties in eating, drinking and swallowing (dysphagia), taste and smell alterations, leading to a reduction in the pleasure of eating, thus a decline in their quality of life [5–7]. It is known that a poor nutritional status during cancer treatment has a negative impact on the frequency and dosage of appropriate treatments, leading to increased length of hospital stay, reduced treatment tolerance, increased treatment side effects, reduced efficacy and treatment delivery or increased risk of mortality [8, 9].

Among cancer treatment side effects, taste and smell alterations are a well-recognised cause of decreased food intake and are reported as being one of the most distressing side effects, along with fatigue, nausea, vomiting and hair loss. They are frequently observed in oncology patients undergoing both radiotherapy and chemotherapy. The self-reported sensory alterations in cancer patients undergoing chemotherapy are high, ranging between 45 and 84% for taste alterations, and can vary according to type of cancer and chemotherapy regimen [6].

Chemosensory alterations include a reduction in smell and taste sensitivity (hyposmia/hypogeusia), abnormally heightened smell or taste sensitivity (hyperosmia/hypergeusia), an absence of smell/taste sensation (anosmia/ageusia) and distortion of normal smell/taste (parosmia/dysgeusia) or a smell/taste perception without an external stimulus (phantosmia/phantogeusia) [10]. Other clinical conditions such as dysphagia, dry mouth, mucositis or nausea/vomiting are also common. Dysphagia is defined as a disorder of swallowing solid, liquid or semi-solid foods [11]. It is common in cancers of the head and neck and can result from obstruction in the mouth, throat or oesophagus and pain on swallowing depending upon tumour location.

Despite their high incidence, taste impairments in cancer patients are not always recognised by healthcare professionals and researchers, leaving important questions unanswered, such as the aetiology of the physiological mechanisms underlying taste alterations [12], and limiting the development of more specific foods to tackle these side effects [13]. Accessibility to a variety of attractive food products is inadequate for cancer patients, with the current clinical nutrition market mainly offering different options for patients at risk of malnutrition, such as food or oral nutritional supplements (ONS). ONS are used when patients are malnourished or are about to undergo potentially toxic therapy. They are designed to address patients’ nutritional requirements and are usually high-energy-dense products, provided in addition to food [13]. The range of products is limited to products such as milkshakes, desserts (e.g. the cream/custard style desserts), soups, purees or texture-modified products.

However, these products do not address the pleasure of eating, nor patients’ food preferences, thus may result in insufficient intake [14], and lead to a negative impact on patients’ quality of life [15]. In the preparation of such products, it is not only their nutritional composition that should be considered, but also patients’ sensory alterations in order to promote the pleasure of eating and prevent malnutrition [13]. The introduction of new products adapted to cancer patients’ needs requires a deep understanding of their problems, and expectations towards foods that are dedicated to them. In addition, the perception of those caring for the patients, such as healthcare professionals and relatives/caregivers, will provide a better understanding of issues encountered by patients.

The goal of this study was to understand the impact of cancer patients’ illness and treatment on their diet and eating habits, their opinions and perceptions of nutritional products currently on the market and their needs and expectations of foods tailored to them. This study is the first to explore these issues from three perspectives: patients’, caregivers’ and healthcare professionals’ (nurses and dieticians) in three countries (Poland, Spain and the UK) with different food traditions, approaches to cancer treatment and availability of food offered to cancer patients.

This research addressed the following questions:

1. What are the non-nutritional functions of food for oncology patients?
2. What are the main psychological and social problems associated with eating for cancer patients?
3. How do the needs, habits, preferences and behaviours related to eating differ among cancer patients, their caregivers and healthcare professionals?
4. What do cancer patients, their caregivers and healthcare professionals believe are the current market gaps in nutrition products aimed at oncology patients?
5. What type of food would cancer patients like or desire from a psychological and social perspective?

## Methods and materials

### Study design and methods

Information related to the needs, habits, preferences and behaviours of cancer patients was collected though a qualitative study. Participants involved in the study included cancer patients, caregivers and healthcare professionals (HCP) from three different European countries: Poland, Spain and the UK. Individual In-Depth Interviews (IDI) were conducted in Poland and the UK and Focus Group Interviews (FGI) with co-creation elements in Spain. Due to the COVID-19 pandemic, all interviews were conducted online. Each interview lasted approximately 2 h. Twenty-one IDIs were conducted in Poland (eight with cancer patients, eight with caregivers and five with nurses), three FGIs in Spain (two with patients and one with caregivers) and 17 IDIs in the UK (seven with patients, four with caregivers and six with dietitians). The method used in each country (IDI or FGI) was dependent on the availability of the participants due to the restrictions in each country because of the COVID-19 pandemic.

The interviews were conducted by three female interviewers with experience in moderating qualitative interviews. At the beginning of each interview, the moderator gave an overview by stating the purpose of the study and explaining the format of the session. Participants were reassured that there were no right or wrong answers and that all conversations were held in confidence. A pre-approved discussion topic guide was used to help direct the discussion. The topics covered included general attitudes towards food and eating, the role of eating and food in the quality of life of cancer patients, food-related issues of cancer patients, perception of existing products for cancer patients, and ideas and expectations for new food solutions. Interviews were semi-structured in nature; hence, the moderator used the discussion guide while having the flexibility to elaborate on important themes that emerged during the interview. The study is part of a larger project (ONCOFOOD)^1^ focused on the development of food solutions for cancer patients.

### Participants

Participants were recruited using different means such as cancer associations and organisations, hospitals and social media depending on the country. The inclusion criteria for cancer patients were: adults aged 18–75 years old, who require or have required texture-modified foods and/or are experiencing or have experienced taste and smell alterations in the last few months as a result of undergoing oncological treatments. Taste and smell alterations were assessed in the recruitment questionnaire according to Amézaga et al. [6]. The inclusion criteria for caregivers included: adults (18 + years old), who are relatives/caregivers and had primary responsibility for shopping and preparing meals for someone who has undergone or is undergoing cancer treatment and currently requires texture-modified foods and/or is experiencing taste and smell alterations as a result of oncological treatments. HCP (clinical and medical oncologists, cancer specialist nurses, speech and language therapists, dietitians, oncology social workers and counsellors) over 18 years old with minimum of 1-year experience in treating oncology patients requiring texture-modified foods and/or experiencing taste and smell alterations were included in the study.

The study was conducted between June and August 2020. In Spain, the study protocol was given a favourable opinion for conduct by the Basque Country Clinical Research Ethics Committee (CEIm-E: Code PI2020032); the Ethics Committee of the Robert Zajonc Institute for Social Studies (03/2020) in Poland and by the University of Reading Research Ethics committee (study number UREC 20/14) in the UK. The study was performed in accordance with the Declaration of Helsinki of Good Clinical Practice guidelines and was recorded as NCT04302792 on the clinical trials database (www.clinicaltrials.gov). Informed consent was obtained from all participants included in the study.

A total of 52 participants took part in the study, 21 from Poland, 17 from UK and 14 from Spain. From these, 25 were cancer patients (21 current patients and four ex-patients), 16 caregivers and 11 HCP. Detailed information on the demographic variables of the participants can be found in Table 1.

**Table 1:** Demographic characteristics of participants (cancer patients, caregivers and healthcare professionals)

	Poland	UK	Spain	Total
Cancer patients (*n*)	8	7	10	25
Age (years) mean + − SD	55.13 (± 10.27)	50.10 (± 13.22)	49.63 (± 14.24)	
Gender *n* (%)				
Men	3 (40)	2 (29)	3 (30)	
Women	5 (60)	5 (71)	7 (70)	
Taste and smell alterations (*n*)	8	7	9	
Dysphagia (*n*)	4	6	4	
Caregivers (*n*)	8	4	4	16
Age (years) mean + − SD	49.12 (± 5.72)	51.50 (± 7.85)	No data	
Gender *n* (%)				
Men	4 (50)	0 (0)	2 (50)	
Women	4 (50)	4 (100)	2 (50)	
Healthcare Professionals—HCP (*n*)	5	6	0	11
Age (years) mean + − SD	No data	39,30 (± 11,41)		
Gender *n* (%)				
Men	0	1 (14)		
Women	5 (100)	6 (86)		

### Data analysis

The interviews were recorded and then transcribed. Interviews conducted in Spain were translated into English. Data analysis from all three countries was done by one member of the research team from Poland who was also one of the moderators, so the interviews from Poland were analysed in Polish (without English translation). The computer-assisted qualitative data analysis software MAXQDA was used to analyse the data. Thematic analysis was used [16], a method of developing qualitative data consisting of the identification, analysis and description of the thematic areas. A mixed approach, both inductive and deductive, was used. Some of the codes were created in advance, based on the literature review and team discussions, while others emerged during the analysis. Extracting some themes from the raw data using an inductive approach was used to avoid conceptual tunnel vision.

**Fig. 1:**
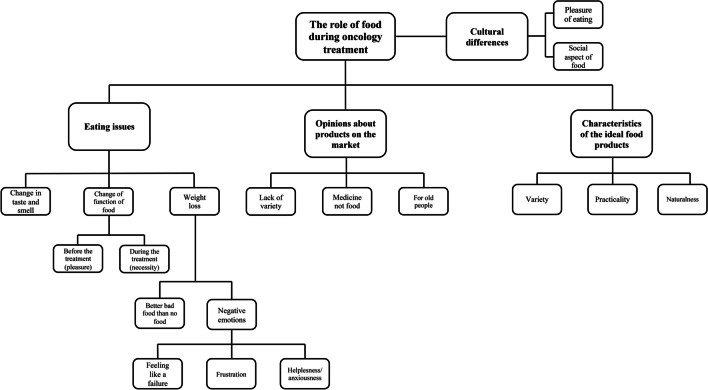
An overview of the codes and themes connected to the role of food during oncology treatment

As a first step, one moderator from Poland reviewed all transcripts. Each transcript was thematically coded individually from this point during the second and third readings respectively. Following this, another researcher reviewed the extracted codes and made initial interpretations by generating themes that captured the essence of the previously identified codes. The researcher then created a list of common themes present in all the interviews. Next, the extracted themes were discussed again with the second researcher conducting the coding in order to achieve consistency. This collaborative process was repeated several times during the analysis. Here, further superordinate and subordinate themes were created, often by collapsing others together, and each theme listed under a superordinate and subordinate category was checked to ensure they were accurately represented. Through this process of repeated analysis and discussion of emerging themes, it was possible to agree on the final themes described as follows: (i) eating issues involved the role of food during treatment and side effects of treatment, (ii) opinions about products on the market focused on the availability of the products and their advantages and disadvantages and (iii) characteristics of the ideal food products featured products not currently available on the market that may meet the needs of the patients. Finally, the researcher conducting the analysis went back to the codes generated earlier to see if any of them are present in some countries and not in others, looking for those that differentiate countries from each other. Codes representing country-specific statements were included in a new theme, containing cultural differences among countries for food perception. An overview of the codes and themes can be found in Fig. 1.

## Results

### Eating issues

The results from the interviews showed that loss of appetite is a common side effect experienced by oncological patients which is related to change in or loss of taste and smell. Participants described foods/drinks as having “paper”, “rotten”, “metallic” and “weird” tastes. This is a problem patients dislike, and they do not have any solutions for. However, because patients understand that eating well is part of their recovery process, they try their best to eat even when they do not find the meal appealing. Patients sometimes try to solve this problem by adding spices or more salt to the food; however, usually, it is without success as the food still tastes unpleasant. The following quote from a UK patient exemplifies such issues:

*When I first started reintroducing foods, rice puddings tasted all right but after a few weeks I could not eat it anymore because it tasted very horrible. Because of damage to the throat, I found tomato soup too spicy, so I was on chicken soup and mushroom soup was one of those things that was OK for a while and then it tasted terrible after a while (…) Nothing I ate tasted right. There is a difference between things tasting horrible and things not tasting right. Six months after my last treatment, my taste is starting to come back, had an orange yesterday and it tasted like an orange. I have also had a Big Mac burger and it tasted alright. It is odd, some things taste right and some things don't.* (Male, patient, 59 y.o., UK).

The study also showed that caregivers are aware of the importance of good nutrition for the patient and place high value on it. From their perspective, the patient’s weight loss is one of the main concerns. In all three countries, we have observed at times an almost “obsessive” approach by caregivers to eating, considering themselves a failure if the person they are caring for is not eating as well as they would expect to. In Poland and the UK, caregivers want their loved ones to eat, even if the food is not the healthiest (for example, too fatty or too sweet). In their opinion, it is more important for a person who is unwell to eat something than to eat a balance diet all the time.

The interviews with caregivers showed clearly that nutrition also has great emotional significance to them. It is not only a way for caregivers to provide adequate nutrients, but also a way of showing their love and care. Therefore, patients’ rejection of caregiver-prepared meals is not only seen as a problem of potential nutrient deficiency, but also (sometimes on a less conscious level) can be perceived emotionally as a sign of the patient’s ungratefulness to the caregiver’s efforts or perhaps a feeling that they had not done their role well enough. Therefore, patients’ problems and complaints about meals (e.g. about lack of taste or bad aftertaste) cause caregivers’ frustration as they feel that they are not appreciated, and their efforts are in vain. They sometimes describe the behaviour of their unwell relatives in the context of food as “acting like children”. They often feel overwhelmed, helpless, anxious and try to do their best to help, but usually without success. This result shows that caregiver’s emotional involvement often does not allow them to perceive patients’ eating problems rationally, but they perceive it emotionally and egocentrically which leads to their frustration. The following quote from a caregiver in Poland demonstrates such frustrations:

*Because many times it's like: Oh, I would eat cauliflower soup, or broth, or this, or that. I say, "Okay, that's cool, it's not that much work. I'm at home all day today, I'll put the pots on and get it done." And then it comes to this: "But I only need a spoonful, just to try it out." I say, "But you wanted it."—"But I don't want it anymore." And then the question: "But why don't you want it, aren't you hungry?"—"I'm hungry, but I want something else." And then it's mostly browsing the fridge, some kind of white cheese sandwich, some kind of yellow cheese sandwich. As for cold cuts, preferably some sirloin, white bologna, oh, yeah—"I could have that day in and day out". Tastes have changed terribly. (…) As if she were pregnant.* (Male, 47 y.o., caregiver, Poland).

The role of food can also change during treatment. Patients, caregivers and HCPs alike pointed to the important function of food during treatment. Food for a cancer patient may change its function from time to time. During cancer treatment, food is a necessity, something that has to be eaten in order to become healthy again and to fuel the body—the pleasure of it is lost. Food becomes part of the healing process, perceived as medicine, with products on the market often looking like medical foods. In the view of a Spanish caregiver:

*And usually in these patients it is forgotten the quality of life, the pleasure for food, because there were people for whom food is a pleasure, and that suddenly with the disease it is lost … they can no longer eat what they ate before …* (Female, 51 y.o., caregiver, Spain).

### Opinions about products on the market

The study showed that while there are products dedicated to cancer patients available on the market, there is a limited variety, they are mostly sweet products and are usually in the form of shakes. The unavailability of other types of food products on the market (e.g. savoury flavours or in formats other than shakes) does have an impact on patients’ pleasure of eating according to the study findings.

In general, patients in all three countries expressed the need for a bigger variety of products for cancer patients. Products dedicated to them will make them feel noticed and valued. Patients were disappointed with what was recommended or prescribed by HCPs as these were mostly milkshakes. Even though these milkshakes are very nutritious and high in calories, they are always very sweet and can have undesirable taste/aftertaste, which was also confirmed by caregivers and HCPs. Cancer patients from all three researched countries expressed their dissatisfaction and feeling of being excluded and neglected by food manufacturers as a group.

Foods for cancer patient’s available on the market are associated with meals for the very sick, old people in the terminal stage of their illness or almost dying (especially in Poland) and patients suffering the hardest side effects of their treatment (in Spain). Therefore, the products available on the market are not perceived as part of everyday diet, rather like another medicine, as something good for a short period, to get an energy and nutritional boost during the most burdensome part of their treatment. However, cancer patients do not want to have a food perceived as a medication as part of their everyday lives, as expressed by two patients from the UK and Spain respectively:

*I see food differently now. I certainly did not see food as pleasure because there was no pleasure in it. I saw food as a necessity. Various people actually told me, you have just got to see food as a medicine and three times a day, you have got to have medicine in the form of food. I am not sure that worked but I got through it. Now that I’ve been off treatment for over a year, I am gradually beginning to see a bit of pleasure in food again. There is the pleasure of being normal, which is very strong, it is the sensation of feeling I am no longer ill because I am eating again, there is the pleasure of being able to share a table with my wife or friends and there is also the pleasure of exploring and experimenting again.* (Male, patient, 60 y.o., UK).

*When I was in the hospital, they gave me a similar product (shakes). I found the taste horrible, and the shakes seemed quite expensive to me. They were usually given to people in the hospital, and I didn't continue taking them after leaving the hospital.* (Woman, patient, 68 y.o., Spain).

### Cultural differences among countries for food perception

There were some differences between countries in terms of importance of the pleasure of eating. In Poland, the pleasure of eating seemed to be crucial—the pleasure of greasy, unhealthy, fried (which is not usually allowed during treatment) meals and alcohol. On the other hand, eating out was not as important as in the UK and Spain. Pleasure of eating in those countries was more connected to its social aspects—especially in Spain where food is a part of social life. Maintaining a social life seems to be very important—social gatherings with family/friends are part of normal life and food is at the centre of it.

*We don't realise how important food is until it is taken from us. This is not just from an energy or nutritional point of view, but the social aspect of eating and just the day-to-day aspect of eating like being able to go and have a cup of tea and cake with friends or go out for a meal. All that is taken away from you because you cannot eat, or you don't want to eat, and it is just huge and very stressful. I spent all day wondering what I could eat and what I wanted to eat.* (Male, patient, 60 y.o., UK).

### Characteristics of the ideal food products

Apart from pleasure, other important characteristics of food for cancer patients in all three countries were variety, practicality and naturalness. As previously stated, most of the products on the market are sweet milkshakes, but patients need more flavours/tastes options to choose from. Products should also be practical. Most patients and caregivers are usually absorbed by all the side effects of the illness on a patient’s life and do not have the time to prepare complicated meals. They also do not have the knowledge and abilities to balance the meals that would reduce taste alterations. For a patient, sometimes even heating up a simple soup could be a challenge.

*There are times during treatment when you wouldn't get out of bed to do anything, not to see what's in the fridge, not to cook it or not to eat. If I can't get up, my husband is working, my kids are in school, what can I do? Then I have looked for services that send me food at home.* (Female, patient, 53 y.o., Spain).

Nutritional composition is crucial while choosing and preparing meals. Most patients are looking for a healthy food product on the market. They care about nutritional composition in terms of fat and, especially, sugar. They try to avoid sugar and “added sugar” products. The healthy and natural characteristics of food are the key factors for them. They look carefully at the ingredients lists of every product to avoid putting additional “chemicals” into their body. This is not limited to the product itself, but also its packaging. Foods should be easy to store and prepared at any time. The healthy lifestyle seemed especially important for the participants from the UK; they seemed to have more knowledge about food and a healthy lifestyle in general than participants from the two other countries.

## Discussion and conclusions

Our research has shown that food and its role are perceived differently by different groups: the patients themselves, their caregivers and medical professionals. First of all, the role of food differs: for medical professionals, the primary function of food is its nutritional value for the cancer patient, while for patients themselves, food has a much larger role beyond its nutritional function. For them, food is part of their social life (especially in Spain) or simply a hedonistic pleasure, so eating problems prevent (or limit) both celebrating food with other loved ones and enjoying it. For this reason, problems with taste or the ability to swallow affect the quality of life of cancer patients. For caregivers, food plays additional roles such as its nutritional function, and for many of them, the food they prepare is a manifestation of their love and care for the patient. Therefore, problems with food (taste changes, difficulty swallowing) leading to rejection of prepared food are a source of great frustration for caregivers. In the situation of the patient’s expressed dislike of the food prepared by the caregiver, the caregiver-patient relationship resembles a parent–child relationship and can even cause the caregiver to become angry with the “whiny” patient. These observations are particularly important in the context that, as previous research has shown, family and friends are a key part of cancer patients’ managing eating problems [17]. Therefore, paying attention to their perspective and the difficulties they feel due to the pressure of being the person “responsible” for the patient is crucial.

The research also showed that in the three countries studied, the range of food products for cancer patients is very limited, making many of the food-related needs of cancer patients impossible to meet. Firstly, most of the products on the market today are sweet drinks with a lack of non-sweet product offerings. Secondly, current products on the market are associated with medication rather than pleasure. Thus, there is a lack of food in a form that more closely resembles normal food (and not just milkshakes).

In addition, there is a lack of products in small packages that can be consumed away from home. Such an offering would provide more opportunities for cancer patients to participate in social activities, such as going out to restaurants with loved ones. One more need expressed directly by cancer patients and their caregivers is for good quality food, based on natural ingredients and with no added sugar. Both patients and their caregivers realise that food for cancer patients should be healthy (especially important for UK respondents, who were far more knowledgeable about nutrition in cancer patients). These findings confirm observations from a previous study of cancer patients in the UK, who prioritised health in nutrition [18]. Unfortunately, in Poland in particular, healthy food is primarily associated with homemade food, which can be a potential barrier to introducing prepared food for cancer patients.

The nutritional composition is important for cancer patients and caregivers. They look carefully at the list of ingredients of every product they buy. They focus on products with no-added sugar that are natural, but also pleasurable. Food becomes part of the healing process, perceived as medicine, with products on the market often looking like medical foods. Secondly, they perceive that there is a gap in the market and cancer patients from all three countries expressed their dissatisfaction on the current market offering. There is an awareness of the few products available on the market, but these products were associated with being ill, and in bad health. Patients desire more flavours, tastes and options to choose from; there is a need for foods with savoury taste rather than sweet (as currently available on the market). The issue of health in the food context from the perspective of the respondents tended to focus on limiting added sugar and the absence of artificial additives, and little attention was paid to other aspects such as protein intake. Patients are recommended to increase their protein intake only in very specific situations such as cachexia (metabolic alteration that provokes involuntary weight loss via skeletal muscle and adipose tissue loss) but not as a regular basis. Considering that the recruited patients suffered from taste and smell alterations, in this case their main concerns were related to the taste, the pleasure of eating and the quality of the products they eat.

When looking at the results several limitations, mostly connected to the method, should be taken into consideration. Firstly, the interviews in two countries (Poland and the UK) were conducted individually whereas focus groups were carried out in Spain. When it comes to talking about food-related practices and the challenges experienced, talking to people with similar experiences can provoke discussion and influence the formulation of new opinions. However, in the case of the described study, the study sample was hard to reach. In addition, the methods used (IDIs or FGIs) were based on the restrictions that we had in each country at the time of the study. Therefore, the main criterion was to conduct the interviews in a way that was accessible to the researchers in the given country—in Spain, where the research team had access to a significant group of patients in the investigator-cooperating hospitals, it was possible to conduct group interviews, whereas in Poland, external recruitment was necessary, making it difficult to conduct group interviews. However, it should be taken into consideration that mixed methods may affect the results obtained, especially those concerning differences between countries.

Based on the results of this study, products addressed to cancer patients suffering from taste and smell alterations products should be tastier (including not only sweet flavours but also salty), natural (no additives and no added sugars) and convenient. Most patients and caregivers are usually absorbed by all the side effects of the illness on a patients’ life and do not have the time to prepare complicated meals. (Foods should be easy to prepare and store; not only for use at home, but also for eating on the go. Ready-to-eat products should be available in small convenient packages that are handy to have in any environment (e.g. when going out with friends and family) enabling patients enjoy the social function of food, which cancer patients miss out on.

New innovative food solutions addressed to cancer patients must be designed considering not only their nutritional needs but also their sensory requirements; capable of alleviating symptoms, preventing malnutrition and promoting the pleasure of eating, emphasising a better quality of life.

## Data Availability

Full transcripts of the interviews are not available for ethical reasons and the privacy of the participants. Excerpts of interviews not published in the article but used for analysis (in the original language) are available upon request.

## Footnotes

1 ONCOFOOD: New food solutions for cancer patients, part of EIT Food project funded by Horizon 2020 research program.
